# Psychometric Properties of the Korean Version of Functioning Assessment Short Test in Bipolar Disorder

**DOI:** 10.1192/j.eurpsy.2023.263

**Published:** 2023-07-19

**Authors:** H. Kang, B.-H. Yoon, H. Yun, Y. Jeong, J. Song

**Affiliations:** Psychiatry, Naju national hospital, Naju, Korea, Republic Of

## Abstract

**Introduction:**

The Functioning Assessment Short Test (FAST) is a relatively specific test for bipolar disorders designed to assess the main functioning problems experienced by patients.

**Objectives:**

FAST includes 24 items assessing impairment or disability in 6 domains of functioning: autonomy, occupational functioning, cognitive functioning, financial issues, interpersonal relationships, and leisure time. It has already been translated into standardized versions in several languages. The aim of this study is to measure the validity and reliability of the Korean version of FAST (K-FAST).

**Methods:**

A total of 209 bipolar disorder patients were recruited from 14 centers in Korea. K-FAST, Young Mania Rating Scale (YMRS), Bipolar Depression Rating Scale (BDRS), Global Assessment of Functioning (GAF) and the World Health Organization Quality of Life Assessment Instrument Brief Form (WHOQOL-BREF) were administered, and psychometric analysis of the K-FAST was conducted

**Results:**

The internal consistency (Cronbach’s alpha) of the K-FAST was 0.95. Test-retest reliability analysis showed a strong correlation between the two measures assessed at a 1-week interval (ICC = 0.97; p < 0.001). The K-FAST exhibited significant correlations with GAF (r=-0.771), WHOQOL-BREF (r=-0.326), YMRS (r=0.509) and BDRS (r=0.598). A strong negative correlation with GAF pointed to a reasonable degree of concurrent validity. Although the exploratory factor analysis showed 4 factors, the confirmatory factor analysis of questionnaires had a good fit for a six factors model (CFI=0.925; TLI=0.912; RMSEA=0.078).

**Table.** Model fit index of confirmatory factor analysis (n=209)

Measure of fit	4-factor model	6-factor model	Acceptable value
χ2/df	2.832	2.267	<3
RMSEA (90% CI)	0.094(0.086-0.102)	0.078 (0.069-0.087)	<0.08
CFI	0.887	0.925	>0.9
TLI	0.873	0.912	>0.9

χ2, chi-square; df, degrees of freedom; RMSEA, root mean square error of approximation; CFI, comparative fit index; TLI, Tucker-Lewis index.

**Conclusions:**

The K-FAST has good psychometric properties, good internal consistency, and can be applicable and acceptable to the Korean context.

**Disclosure of Interest:**

None Declared

